# Deep Learning Detection of Aneurysm Clips for Magnetic Resonance Imaging Safety

**DOI:** 10.1007/s10278-023-00932-8

**Published:** 2024-01-12

**Authors:** Megan Courtman, Daniel Kim, Huub Wit, Hongrui Wang, Lingfen Sun, Emmanuel Ifeachor, Stephen Mullin, Mark Thurston

**Affiliations:** 1 Faculty of Science and Engineering, School of Engineering, Computing and Mathematics, University of Plymouth, Plymouth, PL4 8AA UK; 2 Department of Radiology, Royal Cornwall Hospitals NHS Trust, Truro, TR1 3LJ UK; 3 Department of Radiology, Torbay and South Devon NHS Trust, Torquay, TQ2 7AA UK; 4 Department of Radiology, University Hospitals Plymouth NHS Trust, Plymouth, PL6 8DH UK; 5 Plymouth Institute of Health and Care Research, University of Plymouth, Plymouth, PL4 8AA UK

**Keywords:** Aneurysm clips, Artificial intelligence, CT, Deep learning, MRI, Patient safety

## Abstract

Flagging the presence of metal devices before a head MRI scan is essential to allow appropriate safety checks. There is an unmet need for an automated system which can flag aneurysm clips prior to MRI appointments. We assess the accuracy with which a machine learning model can classify the presence or absence of an aneurysm clip on CT images. A total of 280 CT head scans were collected, 140 with aneurysm clips visible and 140 without. The data were used to retrain a pre-trained image classification neural network to classify CT localizer images. Models were developed using fivefold cross-validation and then tested on a holdout test set. A mean sensitivity of 100% and a mean accuracy of 82% were achieved. Predictions were explained using SHapley Additive exPlanations (SHAP), which highlighted that appropriate regions of interest were informing the models. Models were also trained from scratch to classify three-dimensional CT head scans. These did not exceed the sensitivity of the localizer models. This work illustrates an application of computer vision image classification to enhance current processes and improve patient safety.

**Supplementary Information:**

The online version contains supplementary material available at 10.1007/s10278-023-00932-8.

## Introduction

Screening of patients for aneurysm clips and other metallic devices prior to magnetic resonance imaging (MRI) is vital to ensure that the patient and device can be scanned safely. There have been numerous makes and designs of aneurysm clip over decades [1], many of which have been categorized as MRI conditional. For these particular implants, MRI is not absolutely contraindicated, but the devices need careful prior assessment to ensure that the scan takes place under manufacturer-specified conditions. However, not all historic clips are MRI safe, and even those that are safe in some conditions may not be safe in all conditions [2]. At least one fatality has been caused by the displacement of an aneurysm clip [3]. Safe examination requires review of medical records and co-ordination of multiple experts [4]. Late detection has the potential to result in last-minute cancellations and wasted scanner time. Failure to perform the required checks can result in device dysfunction with potential harm to the patient.

MRI is the standard imaging modality for many conditions. Appropriate screening policies and procedures are essential before permitting entry to the MRI scanner to prevent injury [5]. Best practice is to use referrer and patient questionnaires to identify patients with devices or other issues that need further investigation. Questionnaires are not fail-safe as referrer responses can be unreliable and patient responses are often not available until the day of the scan.

In the last decade, there have been significant advances in AI-based medical image classification due to increased compute power, the open-sourcing of large labelled datasets, and the development of deep learning [6]. Deep learning describes the subset of machine learning which uses layered neural networks to build representations of complicated concepts out of simpler concepts [7]. This negates the need for feature extraction, as required by other methods, and streamlines the preprocessing pipeline [8]. The success of deep learning methods in image classification tasks is well-documented, and for the last decade they have exceeded the performance of many other state-of-the-art classification algorithms [9]. There are now thousands of publications applying deep learning techniques to medical imaging [10].

We describe the design of a deep learning model for the detection of the presence of aneurysm clips in computerized tomography (CT) head scans. The vast majority of patients with aneurysm clips will have had CT head imaging previously performed as part of their treatment, presenting the potential to screen these previous scans as part of an automatic pre-MRI safety check. This would improve MRI safety, reduce last-minute cancellations, and save time and resources.

## Materials and Methods

Ethical approval was granted on 15 October 2019 by HRA and Health and Care Research Wales. Data were obtained from Derriford Hospital, a large teaching hospital with a regional neurosurgery centre serving the South West of the United Kingdom. The study design was retrospective and observational using pre-existing medical image data.

### Subject Inclusion

A database of patients with aneurysm clips was used to identify cases for inclusion in the study. A list of all patients undergoing aneurysm clip surgery was identified from surgical records. The radiology information system (RIS) (Cris, Wellbeing Software) was used to identify all post-surgical CT head examinations for these patients. A custom SQL query was then used to search the RIS for matched controls. For each scan with an aneurysm clip present, a scan with no aneurysm clip present was identified. These control scans were matched according to:

- Scan type
- Age at time of scan, within a window of ± 6 months
- Scan date, within a window of ± 12 months
- Gender

### Image Data Acquisition

Images for the investigations identified on the RIS were downloaded from PACS using dcmtk (OFFIS e.V.) [11]. These studies were anonymized using custom anonymization software based on the Clinical Trials Processor (RSNA MIRC project) [12].

### Ground Truth Confirmation

Manual review of images was performed by two board-certified radiologists to ensure correct labelling. In the event of any disagreement of the correct labels, a third board-certified radiologist reviewed the case to confirm the correct labelling.

### Split

**Fig. 1:**
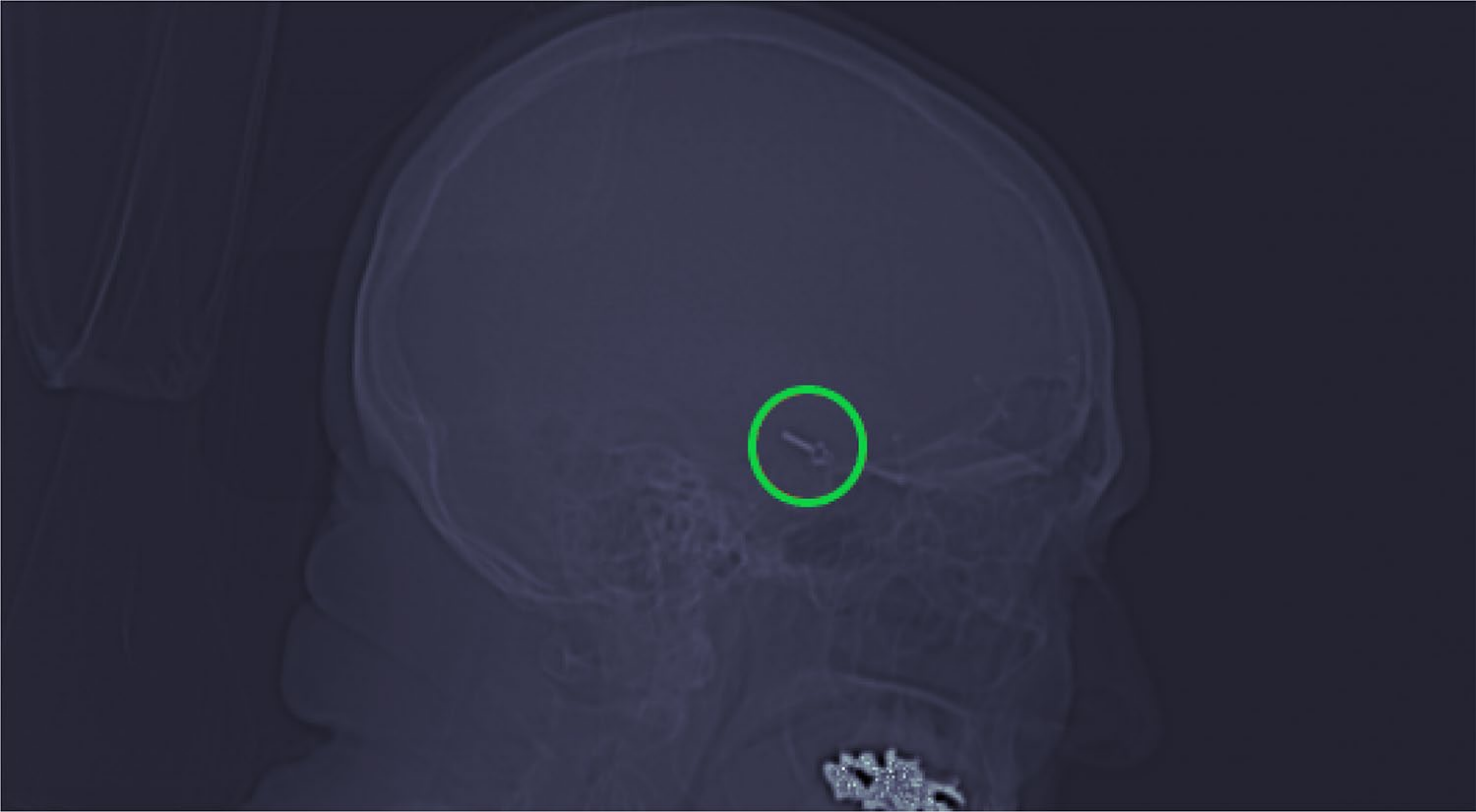
Sagittal localizer with aneurysm clip present, circled

Two sets of images were extracted from the fully curated dataset: a set of localizers and a set of full CT heads. Most CT scan studies begin with one or more localizer scans. These are of poorer quality than full CT scans, but aneurysm clips can often still be clearly seen (Fig. 1). Localizer scans acquired in the same plane were identified automatically using the DICOM tags. From the fully curated dataset, 274 scans were identified which contained sagittal localizers: 136 with aneurysm clips and 134 without. These localizers were randomly divided at a scan level: 28 scans (10%) were reserved as a holdout test set (10 with aneurysm clips and 18 without). The remaining 246 (90%) were used for model development (126 with aneurysm clips and 120 without).

To standardize the full CT head dataset, scans reconstructed using the same kernel were identified automatically using the DICOM tags. From the fully curated dataset, 214 scans were identified which had been reconstructed using a bone kernel: 104 with aneurysm clips and 110 without. These were randomly divided at a scan level: 22 scans (10%) were reserved as a holdout test set (11 with aneurysm clips and 11 without). The remaining 192 (90%) were used for model development (93 with aneurysm clips and 99 without).

For both localizers and full CT heads, fivefold cross-validation was used to develop and assess models, with the data divided into 80% training data and 20% validation data in each fold.

For both types of image, the five developed models were then finally tested on the holdout test set.

### Image Preprocessing

The images were preprocessed before model input by a deterministic automatic pipeline developed in Python using tools from OpenCV [13], SciPy [14] and scikit-image [15]. For the two-dimensional localizer scans, black borders were removed. Pixel values were rescaled between zero and one. Images were cropped to contain the head only, and the bottom of the images removed to exclude the mandible. This optimization was included after the explainability technique revealed that models were being confounded by the presence of fillings, resulting in false positive results. Images were resized to 400 $\times$ 400 pixels.

For the three-dimensional scans, the Hounsfield values were clipped with a level of 2000 and a window of 500 to optimize the visibility of metal. Voxel values were scaled between zero and one. Images were cropped to contain the head only and resized to 256 $\times$ 256 $\times$ 40 voxels.

### Neural Network Architecture

Python-based deep neural networks were built with Keras [16] using the TensorFlow backend [17]. Graphics processing unit hardware acceleration on an NVIDIA GeForce RTX 3080 was used for neural network training. Jupyter Lab [18] was used for model development to enable iterative improvements to be made efficiently.

**Fig. 2:**
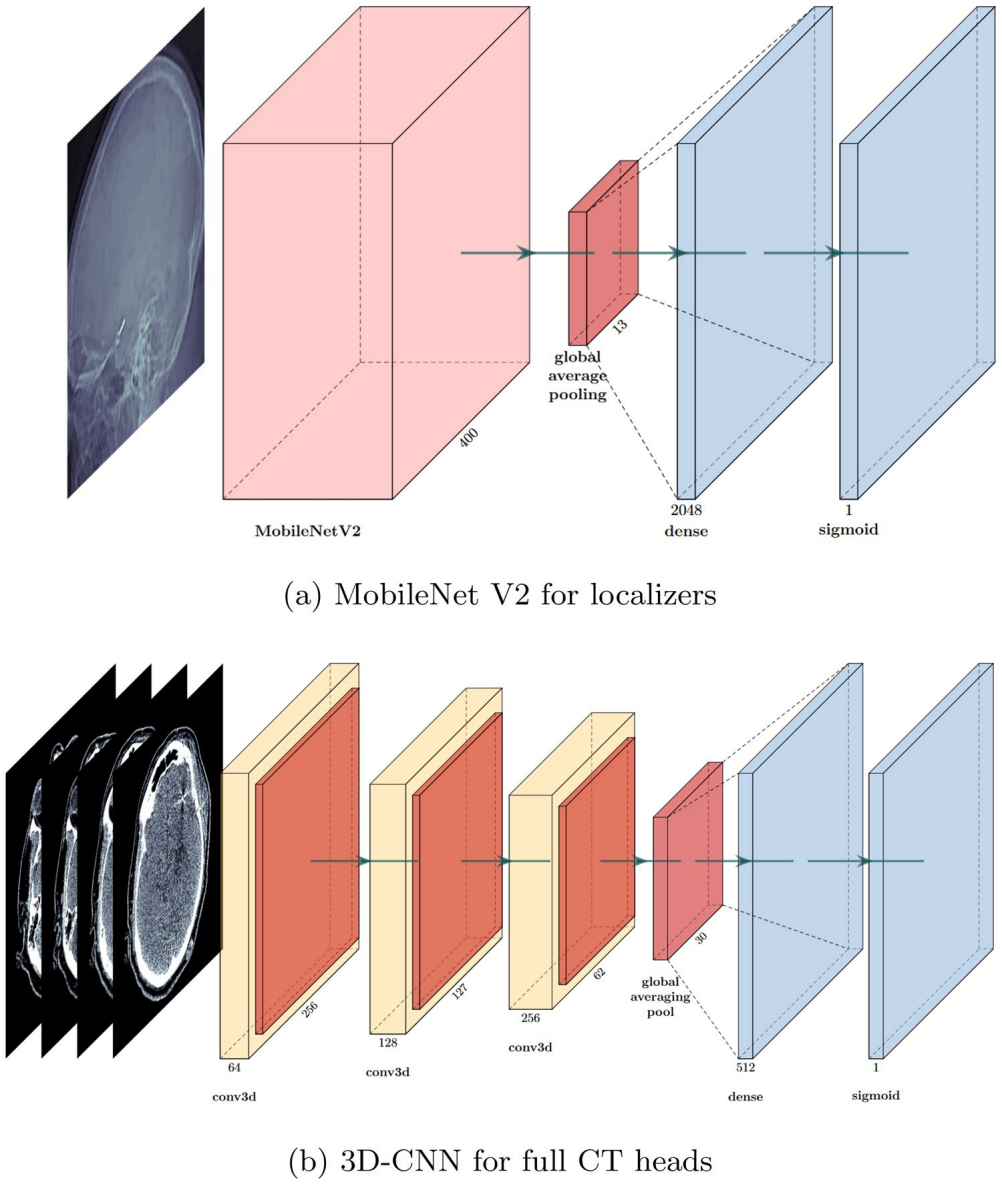
Network architectures

For the classification of the two-dimensional localizer images, a convolutional neural network based on a pre-trained model was selected as a proven choice for computer vision and image classification tasks using transfer learning [10]. Several well-established pre-trained base networks were trialled, including VGG16 [19], Inception V3 [20], Xception [21], DenseNet [22] and MobileNet V2 [23]. Following analysis for each model, MobileNet V2 achieved the greatest performance and was chosen for the final models (Fig. 2a).

For the classification of the three-dimensional CT images, a three-dimensional convolutional neural network was trained from scratch, due to a lack of available pre-trained three-dimensional classification networks [24]. Several different hyperparameter configurations were trialled. Following curve analysis for each iteration, the one which achieved the smallest loss on the validation data was chosen for the final models (Fig. 2b).

### Model Training

The models were trained for a maximum of 100 epochs using stochastic gradient descent with the Adam optimization algorithm (learning rate 0.001) [25]. The binary cross-entropy loss function was utilized. The batch size was 64. The images were augmented with a 50% probability of horizontal flip. Other augmentation methods were trialled, but did not result in any further increase in performance. The models achieving the lowest loss on the validation sets during training were saved using checkpoints.

A classification threshold was then chosen for the models which maximized sensitivity, and therefore minimized the prevalence of false negatives.

### Explainability

SHapley Additive exPlanations (SHAP) were used to explain the 2D models’ predictions. SHAP uses the game theory concept of Shapley values to calculate the contribution of a factor to a machine learning model output [26]. In this case, DeepSHAP was used to calculate and visualize the contribution of individual pixels to the deep learning model’s prediction.

## Results

### Localizer Images

Of the pre-trained base models trialled for the localizer images, MobileNet V2 achieved the greatest mean test Receiver Operating Characteristic (ROC) area under the curve (AUC) and was chosen for the final models. Other base model results are reported in Table 1.

**Table 1:** Performance of different base models for localizer images

Base model	Mean ROC AUC	Parameters	Inference time (ms)	GFLOPS
VGG16	0.84	15,767,361	24.9	97.9
Inception V3	0.95	26,001,185	27.4	21.0
XCeption	0.98	25,059,881	25.5	29.4
DenseNet	0.98	22,258,241	30.7	27.4
MobileNet V2	0.99	4,883,521	26.2	2.0

**Fig. 3:**
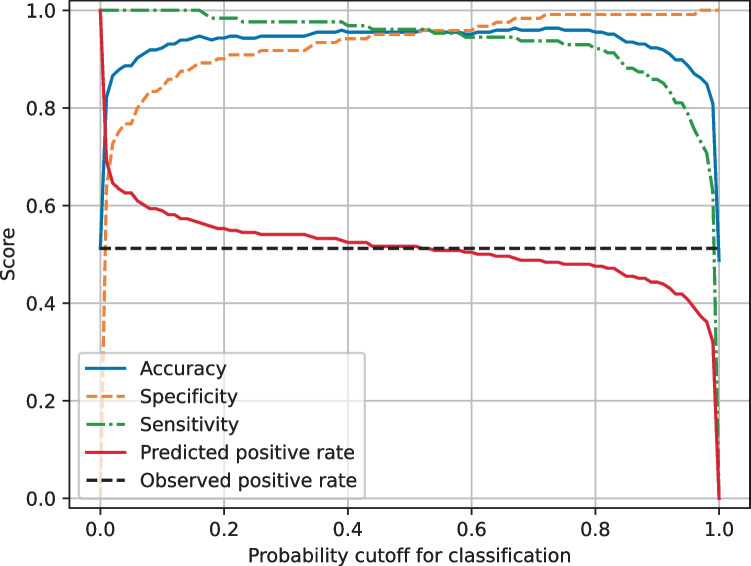
Mean test performance metrics for MobileNet V2 models in training

A classification threshold of 0.16 was chosen to maximize sensitivity whilst maintaining a high accuracy and specificity (Fig. 3). The final models achieved a mean test sensitivity of 100%. Other performance metrics are reported in Table 2.

**Table 2:** Performance metrics for MobileNet V2 models with classification threshold of 0.16

Performance metric	Training mean	Holdout mean
ROC AUC	0.99	1.00
Accuracy	95%	82%
Sensitivity	100%	100%
Specificity	89%	82%

When tested on the holdout test set of 28 localizer images, the final models achieved a sensitivity of 100%. Other performance metrics are reported in Table 2.

#### Incorrectly Classified Examples

The incorrectly classified 2D localizer images were analysed using the SHAP explainability method. In the early stages of the research, this demonstrated the need to remove the mandible from the images, as prior to this removal the models were confounded by the presence of fillings.

**Fig. 4:**
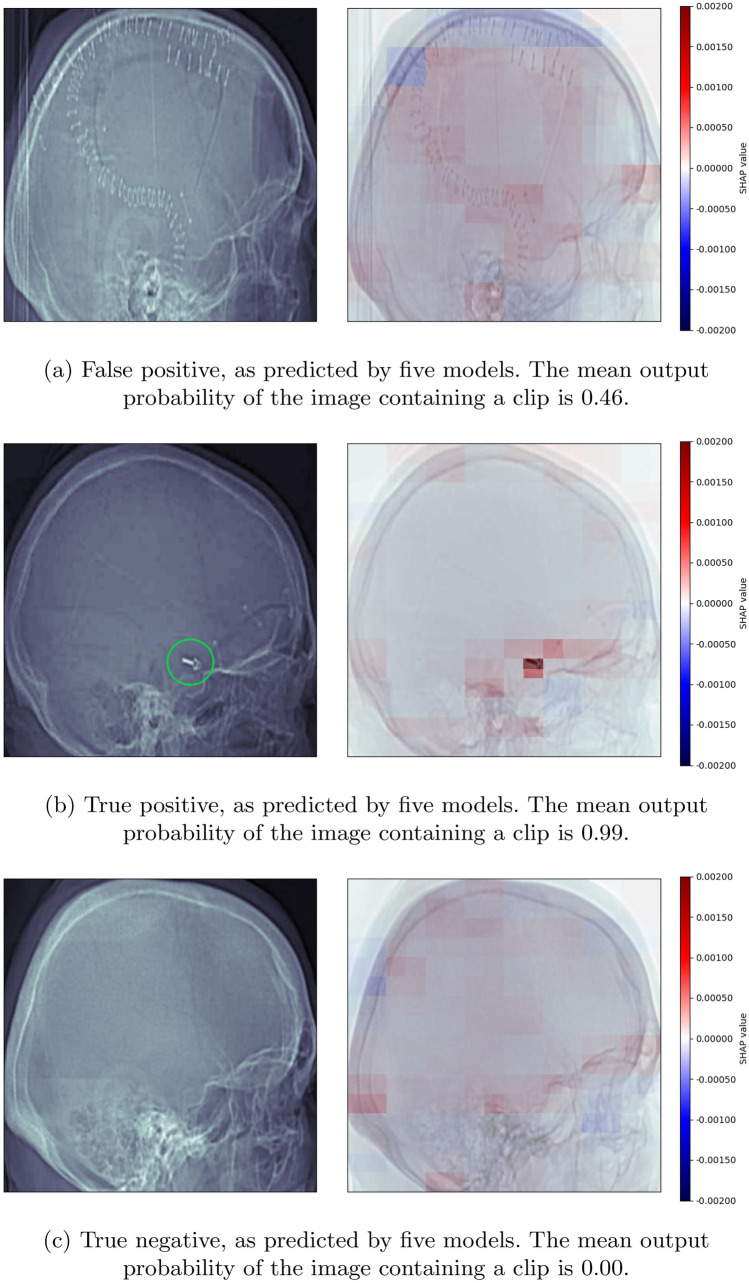
Maps of average SHAP values. Any pixels highlighted in red have contributed to the prediction that an aneurysm clip is present; any pixels highlighted in blue have contributed to the prediction that no aneurysm clip is present. In the case of the true positive, the aneurysm clip has been circled in green for clarity

After the images had been cropped and models developed, the SHAP explainability method was used to analyse the incorrectly classified examples in the holdout test set. Three of the 28 images were incorrectly classified by all five models, and five other images were misclassified by at least one of the models. All of these errors were false positives. The average SHAP maps show that bright areas have contributed to the models’ incorrect predictions, including other metal devices (Fig. 4a).^1^

#### Correctly Classified Examples

The SHAP explainability method was also used to analyse the localizer images that the models classified correctly. Of the 28 images in the holdout test set, 20 were classified correctly by all five models. The average SHAP maps for the true positives show that the pixels containing aneurysm clips contributed positively to models’ correct predictions that a clip is present (Fig. 4b).^2^ The signal is much stronger than the confounding signals in the false positive predictions, and is much stronger than any signal in the true negative predictions where no clip has been detected (Fig. 4c).^3^

### Three-Dimensional CT Images

**Fig. 5:**
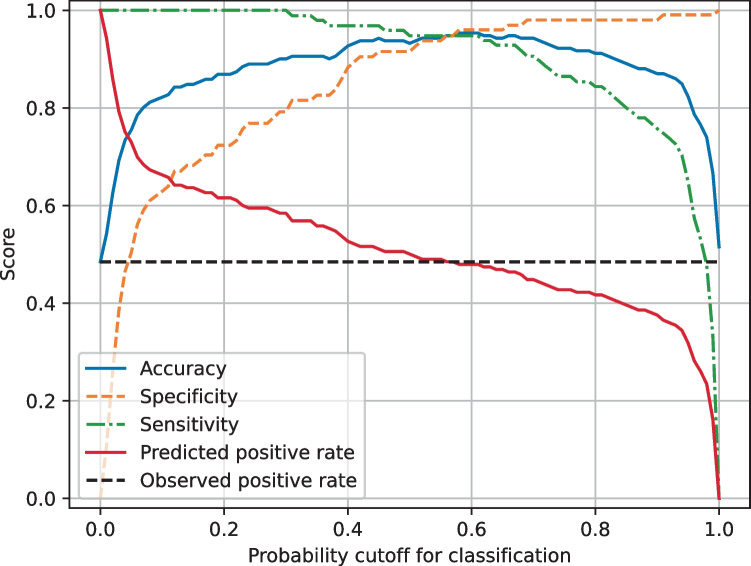
Mean test performance metrics for 3D models in training

After models had been trained on three-dimensional CT images, a classification threshold of 0.30 was chosen to maximize sensitivity whilst maintaining a high accuracy and specificity (Fig. 5). The final models achieved a mean test sensitivity of 96%. Other performance metrics are reported in Table 3.

**Table 3:** Performance metrics for 3D models with classification threshold of 0.30

Performance metric	Training mean	Holdout mean
ROC AUC	0.99	0.96
Accuracy	90%	95%
Sensitivity	100%	96%
Specificity	79%	95%

When tested on the holdout test set of 22 three-dimensional CT images, the final models achieved a mean sensitivity of 96%. Other performance metrics are reported in Table 3. Of the 22 images, 19 were correctly classified by all five models. Of the three images that were incorrectly classified by at least one model, two were false positives and one was a false negative.

## Discussion

Deep learning has previously been used successfully to detect medical implants. Pre-trained convolutional neural networks have been used to detect pacemakers in chest radiographs with 99.67% accuracy [27] and spinal implants in lumbar spine lateral radiographs with 98.7% precision and 98.2% recall [28]. A convolutional neural network trained from scratch has been used to identify dental implants in X-ray images with 94.0% segmentation accuracy and 71.7% classification accuracy [29]. In another application, a segmentation network has been developed to identify orthopedic implants in hip and knee radiographs with 98.9% accuracy and 100% top-three accuracy, exceeding the performance of five senior orthopedic specialists [30].

The successful implementation of deep learning for implant detection is continued in this application, the first to use deep learning to detect aneurysm clips. The trained models exhibit excellent performance for both localizer images and full CT head scans. Both types of model generalize well to the unseen data in the holdout sets and score particularly highly in terms of sensitivity. The sensitivity for the localizer models is 100% in both the training and the holdout data: there are no dangerous false negatives. The computational resources required to run the models are particularly low in the case of the localizer images.

The use of an explainability method is particularly valuable in this application because it demonstrates that the correct parts of the localizer image are informing the models. In general, the positive (red) signal in the images is strongly localized and more observable than the negative (blue) signal, which is weaker and more distributed. This suggests that the models are being positively informed by the presence of aneurysm clips, and are being informed on a more widespread and low level by the absence of aneurysm clips.

As this application is a potential safety tool, the models have been developed and classification thresholds chosen to maximize sensitivity and minimize false negatives. As a result, they are sometimes confounded by other bright areas in the images, making some false positives likely. This could create additional work for a human operator, but it is a preferable error to dangerous false negatives. The heatmaps also demonstrate that other metal devices such as skull flap fixing plates and skin clips can be responsible for false positives (see Supplementary Fig. 1). These are still valuable to detect for MRI safety. Future work could assess these models on a CT head dataset incorporating a wider range of metallic implants, to analyse whether models trained to detect aneurysm clips specifically generalize to metal implant detection more broadly.

It was anticipated that models developed for full CT heads might perform better than models developed for localizer scans, as the aneurysm clip would be presented in three dimensions and in greater detail. However, the sensitivity of the three-dimensional models was slightly poorer. This may have been due to the presence of too much other confounding detail, or may have been due to the models having been trained from scratch rather than taking advantage of pre-learned patterns. Pre-trained networks were used for the localizer scans due to their ready availability for transfer learning in two-dimensional image data. At this time, there is a notable lack of equivalent pre-trained networks available for transfer learning in three-dimensional image data. If pre-trained three-dimensional networks become available in the future, then they might be successfully leveraged in this application.

Future work could consider using an ensemble model. Ensemble methods are considered the state of the art for many machine learning applications, as they harness the power of weaker learners [31]. An ensemble model for this application could incorporate different learning algorithms, as well as bagging or boosting approaches.

### Limitations

The size of the data is a limitation of this research, caused by the rarity of CT scans depicting aneurysm clips. If it were possible to obtain more data this might enable the development of even more accurate models in training, and enable more representative assessment of models in the holdout set. We have mitigated this limitation to an extent by augmenting the training data with horizontal flip, thus artificially increasing the size of the dataset.

Another limitation of this research is the lack of external validation. External validation sets are difficult to obtain as appropriate publicly available databases do not exist. Our research team is in the process of planning and gaining governance clearance for such accessible studies. We have mitigated this limitation as far as possible in this study by reserving an unseen holdout test set. However, these data originate from the same source as the training data, and the metrics reported may not be representative of the models’ performance on data from a different distribution. For example, the balance of the data used in this study is not representative of the typical MRI patient population, in which only a small minority would have aneurysm clips present. An external validation set would allow for more accurate assessment of the models’ capability to generalize to other populations.

## Conclusion

A pre-trained MobileNet V2 neural network achieved high accuracy and 100% sensitivity for the detection of aneurysm clips in CT localizer scans, and the explainability method demonstrated that the network was focusing on appropriate regions of interest in the images. A trained-from-scratch neural network also achieved high accuracy and sensitivity for the detection of aneurysm clips in full CT head scans. This application could be a useful addition to current processes, enabling automatic safety screening for devices in advance of MRI appointments.

### Supplementary Information

Below is the link to the electronic supplementary material.

Supplementary file1 (PDF 10.1 mb)

## Data Availability

Data used in this article are not available due to it being property of the healthcare institution.

## Footnotes

1 See Supplementary Fig. 1 for all false positive average SHAP maps.

2 See Supplementary Fig. 2 for all true positive average SHAP maps.

3 See Supplementary Fig. 3 for all true negative average SHAP maps.

## References

[1] J. T. McFadden, “Magnetic resonance imaging and aneurysm clips: a review,” *Journal of neurosurgery*, vol. 117, no. 1, pp. 1-11, 2012. 10.3171/2012.1.JNS111786 22503120

[2] M. F. Dempsey, B. Condon, and D. M. Hadley, “MRI safety review,” in *Seminars in Ultrasound, CT and MRI*, 2002, vol. 23, no. 5, pp. 392-401. 10.1016/s0887-2171(02)90010-7 12509109

[3] R. P. Klucznik, D. A. Carrier, R. Pyka, and R. W. Haid, “Placement of a ferromagnetic intracerebral aneurysm clip in a magnetic field with a fatal outcome.,” *Radiology*, vol. 187, no. 3, pp. 855-856, 1993. 10.1148/radiology.187.3.8497645 8497645

[4] A. Cunqueiro, M. Lipton, R. Dym, V. Jain, J. Sterman, and M. Scheinfeld, “Performing MRI on patients with MRI-conditional and non-conditional cardiac implantable electronic devices: an update for radiologists,” *Clinical Radiology*, vol. 74, no. 12, pp. 912-917, 2019. 10.1016/j.crad.2019.07.006 31431253

[5] F. G. Shellock and A. Spinazzi, “MRI safety update 2008: part 2, screening patients for MRI.,” *American Journal of Roentgenology*, vol. 191, no. 4, p. 1140, 2008. 10.2214/AJR.08.1038.2 18806156

[6] A. Esteva *et al.*, “Deep learning-enabled medical computer vision,” *npj Digital Medicine*, vol. 4, no. 1, pp. 1-9, 2021. 10.1038/s41746-020-00376-2 PMC7794558 33420381

[7] I. Goodfellow, Y. Bengio, and A. Courville, *Deep learning*. MIT press, 2016.

[8] Y. Bengio *et al.*, “Learning deep architectures for AI,” *Foundations and trends® in Machine Learning*, vol. 2, no. 1, pp. 1-127, 2009.

[9] A. Krizhevsky, I. Sutskever, and G. E. Hinton, “Imagenet classification with deep convolutional neural networks,” *Communications of the ACM*, vol. 60, no. 6, pp. 84-90, 2017.

[10] G. Litjens *et al.*, “A survey on deep learning in medical image analysis,” *Medical image analysis*, vol. 42, pp. 60-88, 2017. 10.1016/j.media.2017.07.005 28778026

[11] OFFIS, “DCMTK,” available via https://dicom.offis.de/dcmtk/. Accessed October 2023.

[12] Radiological Society of North America, Inc., “CTP - The RSNA Clinical Trial Processor,” available via https://mircwiki.rsna.org/index.php?title=MIRC_CTP. Accessed October 2023.

[13] G. Bradski, and A. Kaehler, “OpenCV,” *Dr. Dobb’s journal of software tools*, 3(2), 2000.

[14] P. Virtanen *et al.*, “SciPy 1.0: fundamental algorithms for scientific computing in Python,” *Nature methods*, 17(3), pp.261-272,2020. 10.1038/s41592-019-0686-2 PMC7056644 32015543

[15] S. Van der Walt *et al.*, “scikit-image: image processing in Python,” *PeerJ*, 2, p.e453, 2014. 10.7717/peerj.453 PMC4081273 25024921

[16] F. Chollet and others,“ Keras.” 2015.

[17] M. Abadi *et al.*, “TensorFlow: Large-Scale Machine Learning on Heterogeneous Systems.” 2015.

[18] T. Kluyver *et al.*, “Jupyter Notebooks - a publishing format for reproducible computational workflows,” in Positioning and Power in Academic Publishing: Players, Agents and Agendas, 2016, pp. 87-90.

[19] K. Simonyan and A. Zisserman, “Very deep convolutional networks for large-scale image recognition,” arXiv preprint arXiv:1409.1556, 2014.

[20] C. Szegedy, V. Vanhoucke, S. Ioffe, J. Shlens, and Z. Wojna, “Rethinking the inception architecture for computer vision,” in *Proceedings of the IEEE conference on computer vision and pattern recognition*, 2016, pp. 2818-2826.

[21] F. Chollet, “Xception: Deep learning with depthwise separable convolutions,” in *Proceedings of the IEEE conference on computer vision and pattern recognition*, 2017, pp. 1251-1258.

[22] G. Huang, Z. Liu, L. Van Der Maaten, and K. Q. Weinberger, “Densely connected convolutional networks,” in *Proceedings of the IEEE conference on computer vision and pattern recognition*, 2017, pp. 4700-4708.

[23] M. Sandler, A. Howard, M. Zhu, A. Zhmoginov, and L.-C. Chen, “Mobilenetv2: Inverted residuals and linear bottlenecks,” in *Proceedings of the IEEE conference on computer vision and pattern recognition*, 2018, pp. 4510-4520.

[24] S. Chen, K. Ma, and Y. Zheng, “Med3d: Transfer learning for 3d medical image analysis,” arXiv preprint arXiv:1904.00625, 2019.

[25] D. P. Kingma and J. Ba, “Adam: A method for stochastic optimization,” arXiv preprint arXiv:1412.6980, 2014.

[26] S. M. Lundberg and S.-I. Lee, “A Unified Approach to Interpreting Model Predictions,” in *Advances in Neural Information Processing Systems* 30, I. Guyon, U. V. Luxburg, S. Bengio, H. Wallach, R. Fergus, S. Vishwanathan, and R. Garnett, Eds. Curran Associates, Inc., 2017, pp.

[27] M. D. V. Thurston, D. H. Kim, and H. K. Wit, “Neural network detection of pacemakers for MRI safety,” *Journal of Digital Imaging*, vol. 35, no. 6, pp. 1673-1680, 2022. 10.1007/s10278-022-00663-2 PMC9712856 35768751

[28] H.-S. Yang, K.-R. Kim, S. Kim, and J.-Y. Park, “Deep learning application in spinal implant identification,” *Spine*, vol. 46, no. 5, pp. E318-E324, 2021. 10.1097/BRS.0000000000003844 33534442

[29] A. Kohlakala, J. Coetzer, J. Bertels, and D. Vandermeulen, “Deep learning-based dental implant recognition using synthetic X-ray images,” *Medical & Biological Engineering & Computing*, vol. 60, no. 10, pp. 2951-2968, 2022. 10.1007/s11517-022-02642-9 PMC9385426 35978215

[30] R. Patel, E. H. Thong, V. Batta, A. A. Bharath, D. Francis, and J. Howard, “Automated identification of orthopedic implants on radiographs using deep learning,” *Radiology: Artificial Intelligence*, vol. 3, no. 4, 2021.4765-4774. 10.1148/ryai.2021200183 PMC8328106 34350407

[31] O. Sagi and L. Rokach, “Ensemble learning: A survey,” *Wiley Interdisciplinary Reviews: Data Mining and Knowledge Discovery*, vol. 8, no. 4, p. e1249, 2018.

