# Supplementary material for: Deep Learning Detection of Aneurysm Clips for Magnetic Resonance Imaging Safety
Source: J Imaging Inform Med. 2024 Jan 12;37(1):72–80. doi: 10.1007/s10278-023-00932-8 (PMC10976925; doi:10.1007/s10278-023-00932-8)
Supplement: Supplementary file 1 — Supplementary file1 (PDF 10.1 mb) [file 10278_2023_932_MOESM1_ESM.pdf]

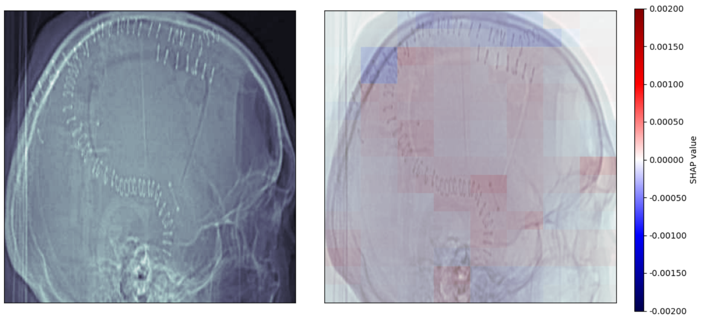

(a) False positive, as predicted by five models. The mean output probability of the image containing a clip is 0.46.

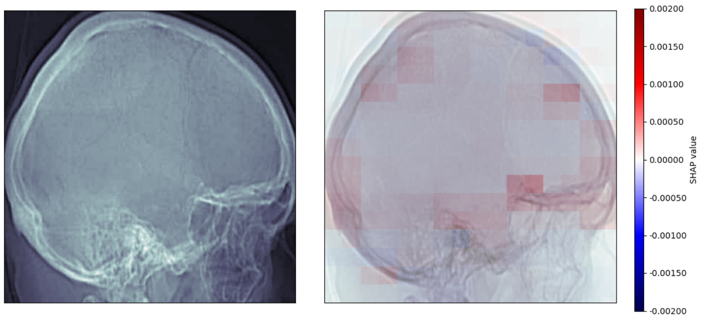

(b) False positive, as predicted by five models. The mean output probability of the image containing a clip is 0.45.

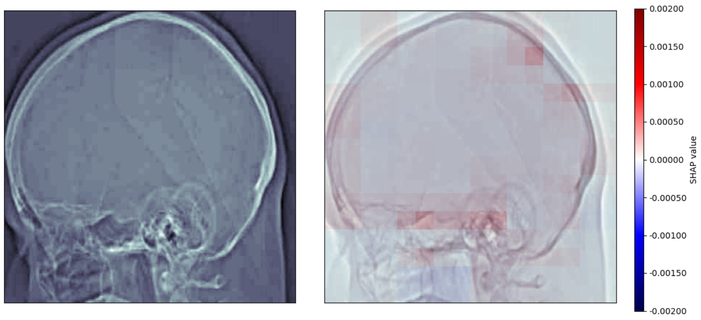

(c) False positive, as predicted by five models. The mean output probability of the image containing a clip is 0.27.

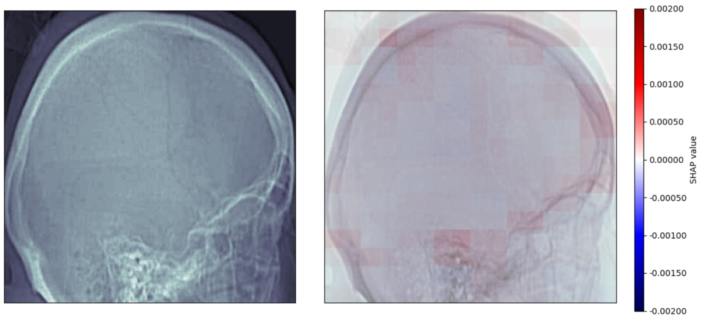

(d) False positive, as predicted by one model. The mean output probability of the image containing a clip is 0.10.

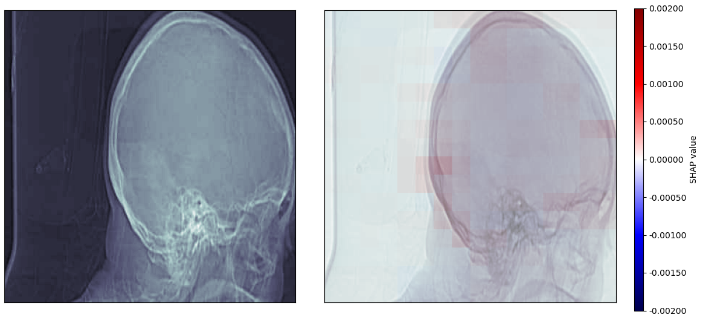

(e) False positive, as predicted by one model. The mean output probability of the image containing a clip is 0.17.

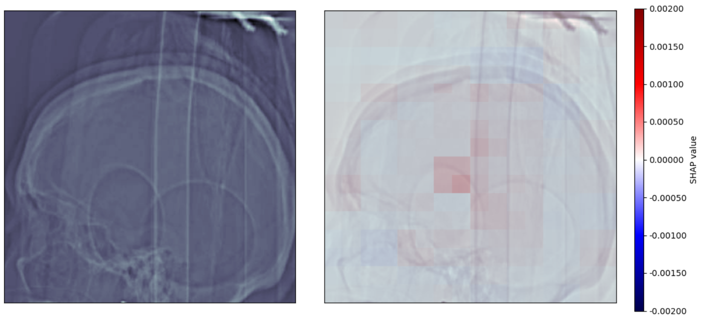

(f) False positive, as predicted by one model. The mean output probability of the image containing a clip is 0.10.

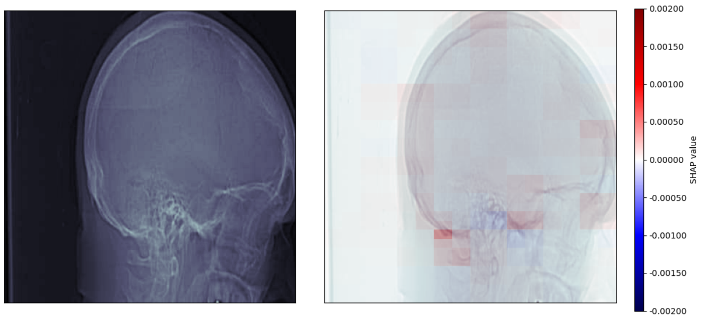

(g) False positive, as predicted by two models. The mean output probability of the image containing a clip is 0.15.

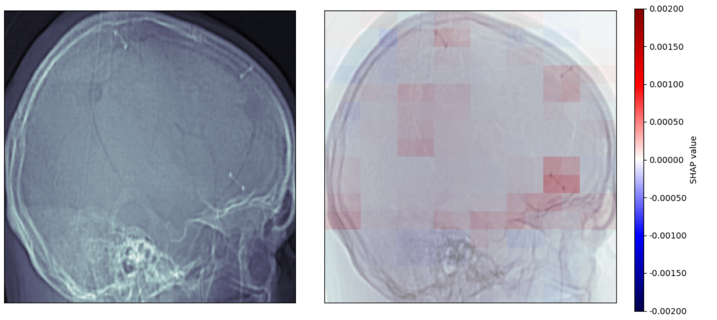

(h) False positive, as predicted by four models. The mean output probability of the image containing a clip is 0.30.

Supplementary figure 1: Maps of average SHAP values for false positive predictions. Any pixels highlighted in red have contributed to the prediction that an aneurysm clip is present; any pixels highlighted in blue have contributed to the prediction that no aneurysm clip is present.

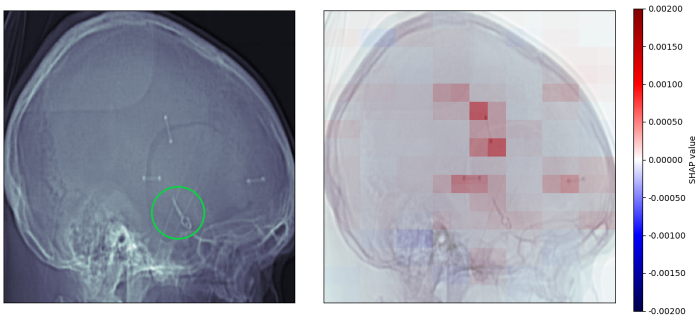

(a) True positive, as predicted by five models. The mean output probability of the image containing a clip is 1.00.

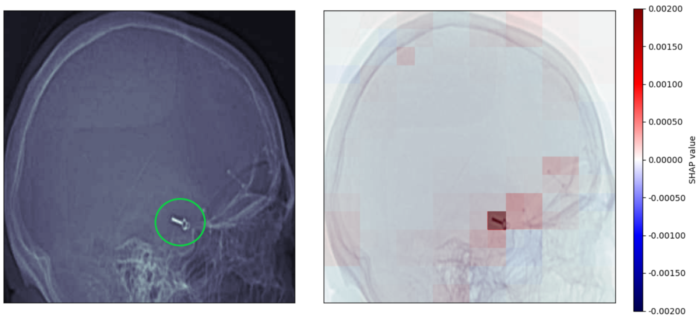

(b) True positive, as predicted by five models. The mean output probability of the image containing a clip is 0.98.

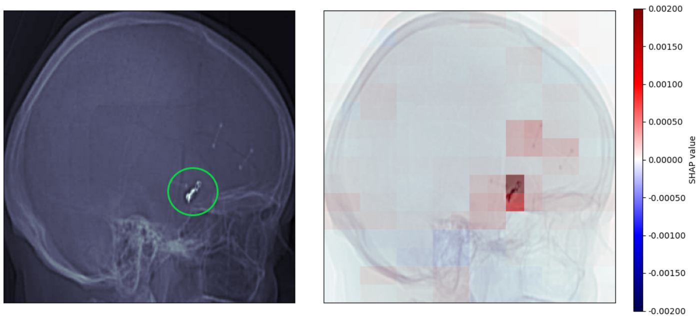

(c) True positive, as predicted by five models. The mean output probability of the image containing a clip is 1.00.

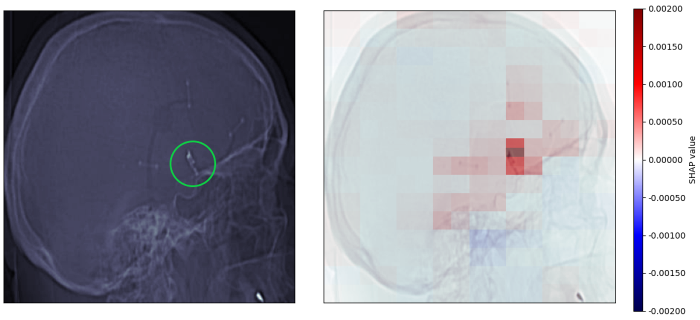

(d) True positive, as predicted by five models. The mean output probability of the image containing a clip is 1.00.

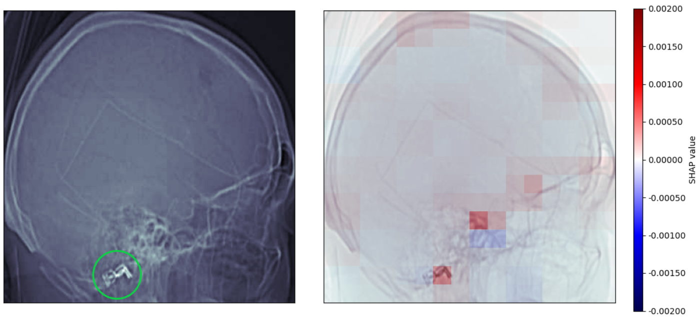

(e) True positive, as predicted by five models. The mean output probability of the image containing a clip is 0.84.

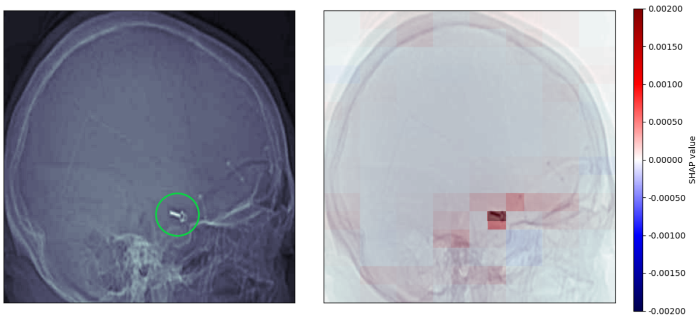

(f) True positive, as predicted by five models. The mean output probability of the image containing a clip is 0.99.

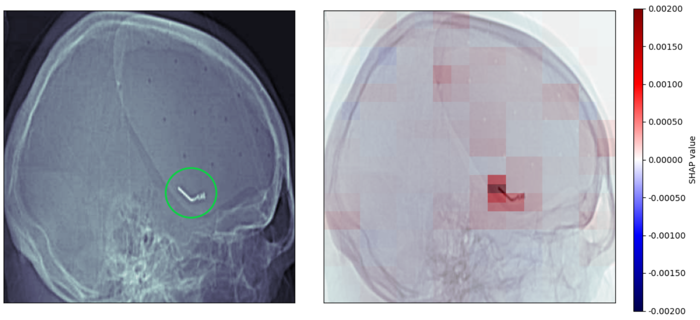

(g) True positive, as predicted by five models. The mean output probability of the image containing a clip is 1.00.

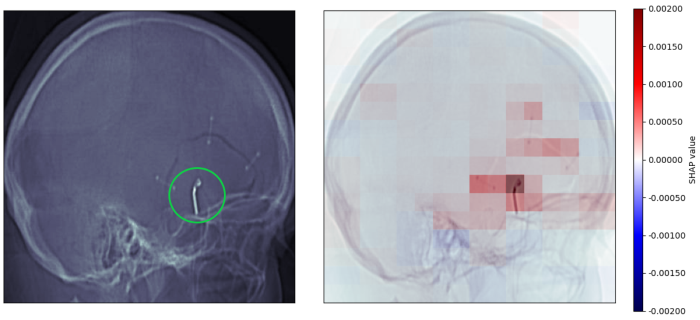

(h) True positive, as predicted by five models. The mean output probability of the image containing a clip is 1.00.

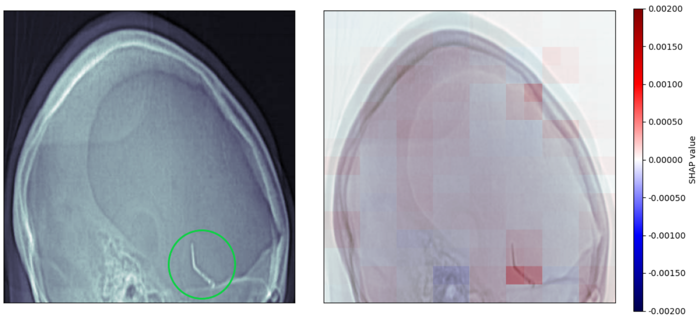

(i) True positive, as predicted by five models. The mean output probability of the image containing a clip is 0.75.

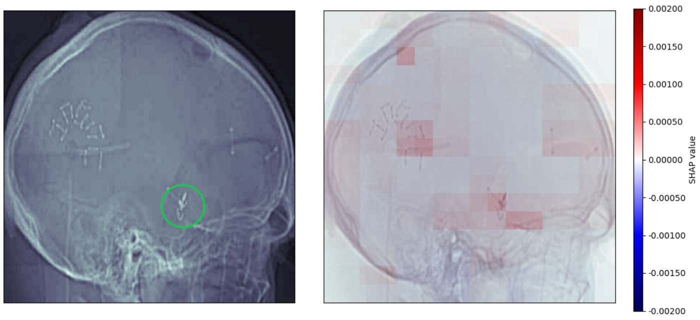

(j) True positive, as predicted by five models. The mean output probability of the image containing a clip is 0.99.

Supplementary figure 2: Maps of average SHAP values for true positive predictions. Aneurysm clips are circled in green. Any pixels highlighted in red have contributed to the prediction that an aneurysm clip is present; any pixels highlighted in blue have contributed to the prediction that no aneurysm clip is present.

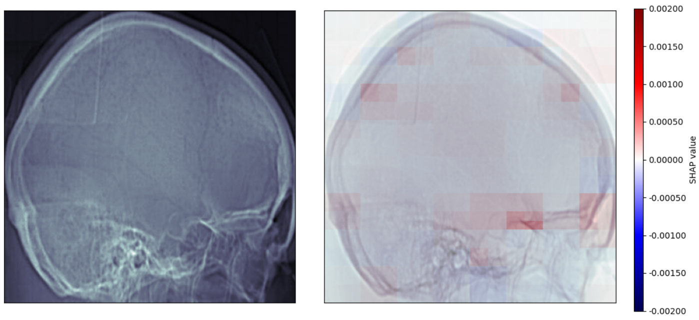

(a) True negative, as predicted by five models. The mean output probability of the image containing a clip is 0.00.

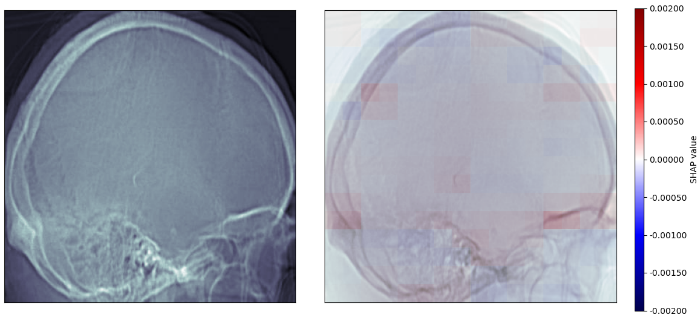

(b) True negative, as predicted by five models. The mean output probability of the image containing a clip is 0.00.

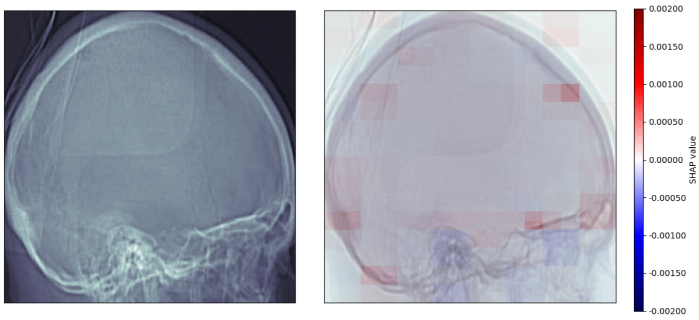

(c) True negative, as predicted by five models. The mean output probability of the image containing a clip is 0.00.

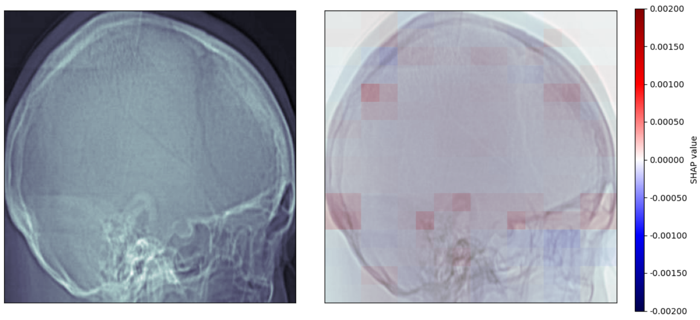

(d) True negative, as predicted by five models. The mean output probability of the image containing a clip is 0.00.

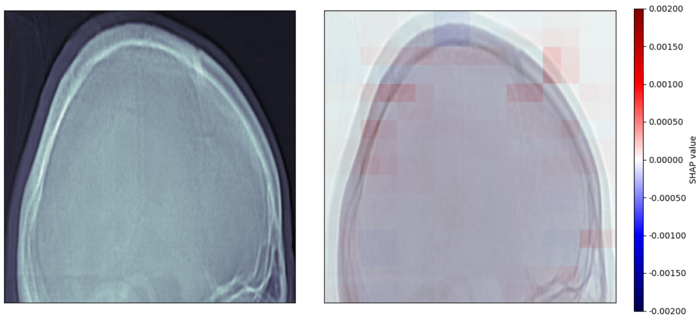

(e) True negative, as predicted by five models. The mean output probability of the image containing a clip is 0.00.

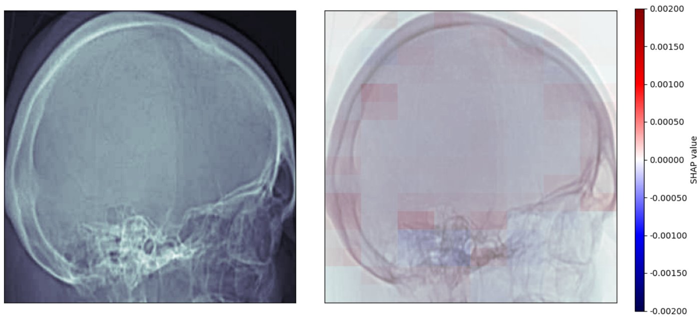

(f) True negative, as predicted by five models. The mean output probability of the image containing a clip is 0.01.

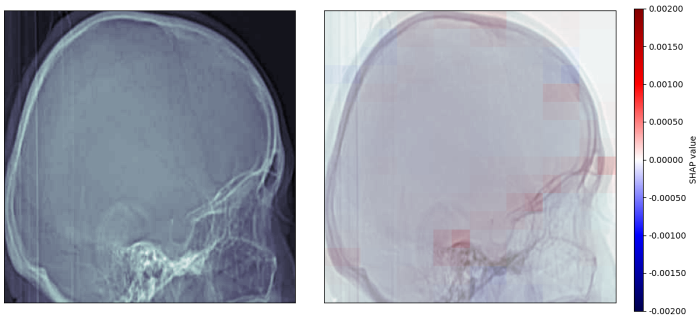

(g) True negative, as predicted by five models. The mean output probability of the image containing a clip is 0.00.

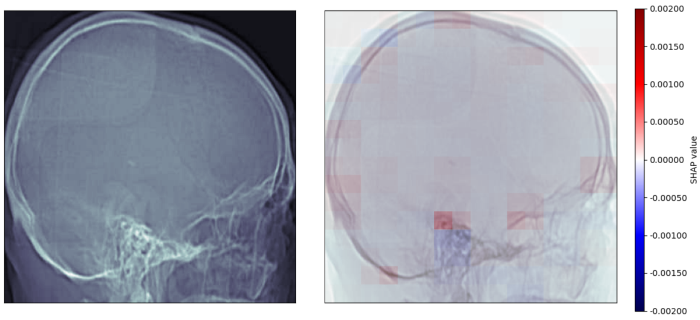

(h) True negative, as predicted by five models. The mean output probability of the image containing a clip is 0.00.

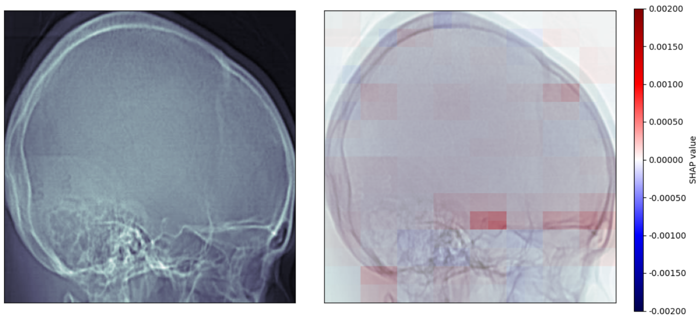

(i) True negative, as predicted by five models. The mean output probability of the image containing a clip is 0.01.

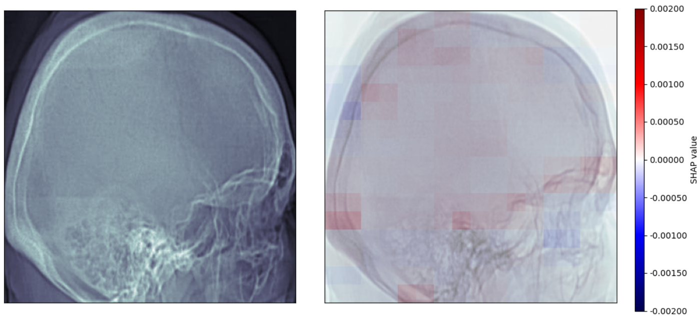

(j) True negative, as predicted by five models. The mean output probability of the image containing a clip is 0.00.

Supplementary figure 3: Maps of average SHAP values for true negative predictions. Any pixels highlighted in red have contributed to the prediction that an aneurysm clip is present; any pixels highlighted in blue have contributed to the prediction that no aneurysm clip is present.
